# Negative Density Dependence Regulates Two Tree Species at Later Life Stage in a Temperate Forest

**DOI:** 10.1371/journal.pone.0103344

**Published:** 2014-07-24

**Authors:** Tiefeng Piao, Jung Hwa Chun, Hee Moon Yang, Kwangil Cheon

**Affiliations:** 1 Institute of Soil and Water Conservation, Northwest A&F University, Yangling, China; 2 Division of Forest Ecology, Department of Forest Conservation, Korea Forest Research Institute, Seoul, Republic of Korea; DOE Pacific Northwest National Laboratory, United States of America

## Abstract

Numerous studies have demonstrated that tree survival is influenced by negative density dependence (NDD) and differences among species in shade tolerance could enhance coexistence via resource partitioning, but it is still unclear how NDD affects tree species with different shade-tolerance guilds at later life stages. In this study, we analyzed the spatial patterns for trees with *dbh* (diameter at breast height) ≥2 cm using the pair-correlation *g*(*r*) function to test for NDD in a temperate forest in South Korea after removing the effects of habitat heterogeneity. The analyses were implemented for the most abundant shade-tolerant (*Chamaecyparis obtusa*) and shade-intolerant (*Quercus serrata*) species. We found NDD existed for both species at later life stages. We also found *Quercus serrata* experienced greater NDD compared with *Chamaecyparis obtusa*. This study indicates that NDD regulates the two abundant tree species at later life stages and it is important to consider variation in species' shade tolerance in NDD study.

## Introduction

Tree populations are often thought to be regulated by negative density dependence (NDD) that can occur during several life stages, because higher conspecific density can impair performance due to stronger intraspecific competition for resources, more susceptibility to pathogens and easier detection by herbivores [1]. Therefore, NDD can result in lower growth and survival of local abundant species [2]. The supporting results include observational [2]–[8] and experimental studies [9]–[13] that take place in tropical [14]–[17], subtropical [18] and temperate forest [19]–[22]. Johnson et al. [23] even further concluded that NDD explains the latitudinal gradient of tree species richness.

A challenge for studies of NDD is caused by lagged effects [24] which are non-fatal effects on individuals during one life stage that influence the growth and mortality rate during the following life stage [25]. Mortality patterns in seedlings can normally be analyzed through direct observation, due to their high susceptibility to natural enemies and environmental stress. However, for established trees that have lower mortality rates, long time-scales observation is required to analyze mortality pattern because NDD may not be strong enough to induce immediate mortality, but instead cause limited growth over short time-scales [24]. This may explain why few studies have been implemented to test NDD on trees at later life stages [4], [26]–[29]. Peters [4] found patterns consistent with NDD for saplings and trees of >75% of the species tested at sites in Pasoh, Malaysia, and BCI, Panama. Lan et al. [29] found saplings of 83.2% of species have a aggregated pattern, whereas adults of 96.2% species have a random distribution, implying that NDD can make the spatial pattern of tropical trees more regular with time. Evidence have shown that different mechanisms shape plant communities at different life stages [30]–[32], therefore, it is important to evaluate NDD at later life stages. One robust way to indirectly examine NDD on the long history of a forest is to analyze the changes in spatial patterns of trees at different life stages [20], [33], [34]. This is possible because the regulating mechanisms that are operating in a forest should have left a detectable spatial signature [35]. If NDD is prevalent and works constantly during the whole life-cycle of trees, the following signatures should be left on the spatial pattern of trees: trees survive better with fewer conspecific neighbors and the clustering of conspecific trees declines with time [36], [37]. However, the above predictions can be obscured by habitat heterogeneity [33], because the performance of individuals can be greatly influenced by the availability of environmental resources [38]–[40], and the functional traits that are involved in plant-enemy interactions might be altered by abiotic and biotic factors [41]. This change in performance with environment can affect a population's susceptibility to herbivory to better link NDD with habitat heterogeneity [18]. For example, one population may show NDD pattern if resource availability is low and reduces the ability of individuals to survive herbivore damage, whereas one population with ample resources may not show the same effect. Therefore, habitat heterogeneity must be considered in NDD study. However, this is made difficult by the fact that many environmental covariates are difficult to quantify [20]. Getzin et al. [33] used a simple method to solve this problem. By utilizing the spatial pattern of adult trees (*dbh*≥10 cm), they factored out large-scale habitat heterogeneity and were able to detect NDD in western hemlock populations.

Species varies in the strength of NDD, arise from differences in morphological, physiological and allocational traits [42]. Shade-tolerant, slow-growing species may experience less NDD than shade-intolerant, fast-growing species [43], [44]. One of the possible mechanisms behind the differences is that shade-tolerant species tend to have higher tissue density and carbohydrate storage for increasing tolerance to herbivores [43] and recover from damage compared with shade-intolerant species [45], [46]. Previous studies have shown that seedling shade tolerance is negatively correlated with disease susceptibility [40] and depression of growth and survival [47]. Also, in a tropical forest study, Comita and Hubbell [7] found seedling survival tended to be positively correlated with species abundance before controlling for shade tolerance. After controlling for shade tolerance, they found a significant negative relationship between seedling survival and species' shade tolerance. Therefore, shade tolerance should be accounted for in NDD study. However, few NDD studies implemented at later life stages considered the variation in shade tolerance among species.

The aim of this study is to examine NDD at later life stage for the most abundant shade-tolerant and shade-intolerant species at Keumsan long-term ecological research plot (Keumsan LTER) which is located in South Korea. The study was implemented by testing whether the intensity of aggregation was decreased from saplings to juveniles. The large-scale habitat heterogeneity was accounted for by utilizing the spatial pattern of adult trees. We first determined whether saplings exhibit additional aggregated patterns relative to adults, then tested whether the additional aggregation decreases from saplings to juveniles (the definitions of “sapling”, “juvenile” and “adult” are provided in the Methods). Finally, we compared whether the shade-intolerant species experiences stronger NDD compared with the shade-tolerant species across size classes.

## Materials and Methods

### Study site

Our 1-ha (100×100 m) study site, Keumsan LTER (34°30′N, 127°59′E), is located in the southern area of the Hallyeohaesang National park in Gyeongsangnam-do, South Korea. The area is located in a warm temperate forest zone. The annual mean temperature and precipitation were 14.0°C and 2,180.0 mm, respectively, and the mean monthly temperature ranged from −1.5°C (January) and 25.6°C (August) in 2011 [48]. The study site is a secondary forest which had suffered great damage during the Korean War (1950–1953). Thereafter, the forest has been well conserved and underwent secondary succession [49]. The Keumsan LTER is located at a 360–430 m hillside elevation with slopes ranging from 12°–28°, and is influenced by habitat heterogeneity such as edaphic gaps (e.g. gravels or wet drainage sites) [50]. The most abundant species are *Quercus serrata*, *Chamaecyparis obtusa*, *Styrax japonica*, *Acer pseudo-sieboldianum*, *Carpinus tschonoskii* and *Stewertia pseudo-camellia*. The Hallyeohaesang National Park is owned and managed by the state and its government and the location including our study area is not privately-owned. No specific permits were required for the described field studies. The field studies did not involve endangered or protected species.

### Data collection

The Keumsan LTER was established in 2000. All woody stems ≥2 cm *dbh* were mapped, measured, identified to species, and tagged. The plot was recensused in 2006 and 2011. In the 2011 census, we documented 2,412 free-standing live individuals ≥2 cm *dbh* belonging to 20 families, 22 genera and 35 species. In this paper, we used data on live trees ≥2 cm *dbh* from the 2011 census for spatial point pattern analysis.

We selected *Chamaecyparis obtusa* (CHOB) and *Quercus serrata* (QUSE) as focal species. *Chamaecyparis obtusa*, a slow-growing, late successional species [51], [52], was the most abundant shade-tolerant tree species at the site, and accounted for 32.5% of the total individuals and 11.3% of total basal area (Table 1); *Quercus serrata*, an early successional species [53], was the most abundant shade-intolerant tree species at the site, and accounted for 10.7% of the total individuals and 53.9% of total basal area (Table 1). Young trees of the two species were usually found under different light conditions: QUSE were often found in forest gaps, while CHOB were often found in shaded areas.

**Table 1:** Species composition in the Keumsan LTER.

Species name	No. Ind. (%)	Basal area (m^2^) (%)	Shade tolerance
*Chamaecyparis obtusa*	784 (32.5)	3.68 (11.3)	Shade-tolerant
*Quercus serrata*	257 (10.7)	17.6 (53.9)	Shade-intolerant
*Acer pseudo-sieboldianum*	253 (10.5)	1.08 (3.3)	Shade-tolerant
*Styrax japonicas*	138 (5.7)	1.19 (3.6)	Mid-tolerant
*Stewartia pseudo-camellia*	125 (5.2)	0.62 (1.9)	Shade-tolerant
*Carpinus cordata*	97 (4)	0.42 (1.3)	Shade-intolerant
*Sapium japonicum*	98 (4)	0.26 (0.8)	Shade-tolerant
*Cornus kousa*	92 (3.8)	0.33 (1)	Mid-tolerant
*Carpinus tschonoskii*	93 (3.8)	2.43 (7.5)	Shade-tolerant
*Acer palmatum*	79 (3.3)	0.22 (0.7)	Shade-tolerant
*Rhododendron schlippenbachii*	77 (3.2)	0.06 (0.2)	Mid-tolerant
*Fraxinus sieboldiana*	51 (2.1)	0.11 (0.3)	Mid-tolerant
Others	268 (11.1)	4.62 (14.2)	-
Total	2412	32.62	-

The figures in brackets show the percentage of the total amounts. The table is sorted in descending order according to the number of individuals.

All individuals of the two species were divided into three size classes as an indication for life history stages: sapling, juvenile and adult (Note the terminology differ from their original meanings, they were used only for classifying different size classes of trees and facilitating spatial point pattern analyses). For CHOB: saplings with *dbh* ranging from 2 to 5 cm, juveniles with *dbh* ranging from 5 to 10 cm and adults with *dbh* ≥10 cm; for QUSE: saplings with *dbh* ranging from 2 to 20 cm, juveniles with *dbh* ranging from 20 to 30 cm and adults with *dbh* ≥30 cm. *Dbh* cut-offs were selected to ensure adequate sample sizes for the spatial point pattern analysis. Both species had more than 60 individuals in each size class (Table S1 and S2). Because using different cut-offs may lead to different results, we also used all other cut-offs (with an increment of 1 cm) (on the condition that more than 60 individuals are included in each life stage) (Table S1 and S2) to implement the analyses.

### Data analysis

In recent studies, spatial point pattern analysis which compares patterns of trees in different size classes has been shown to be an effective approach for testing NDD on established trees [18], [20], , with the assumption that populations of established trees are in an equilibrium stage. We utilized the bivariate pair correlation *g*(*r*) function [55], [56] to implement the analyses. The *g*(*r*) function is the probability density function of the broadly used Ripley's *K*(*r*) function [57], which is calculated as:
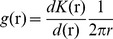



*K(r)* is the cumulative distribution function of the expected number of points of a pattern within the whole circle of a given radius *r* around a typical point of the pattern divided by the intensity λ (points per unit area) of the pattern. *K*(*r*) is a cumulative distribution function where *K*(*r*) is the expected number of other points of a pattern within the whole circle of a given radius *r* around a typical point of the pattern divided by the intensity λ of the pattern. In contrast, the *g*(*r*) is a non-cumulative distribution function in which *g*(*r*) is the expected density of other points in a ring of a given distance *r* around a focal point divided by the intensity λ of the pattern [55]. The *g*(*r*) function can be used to estimate the strength of aggregation at specific scales. If *g*(*r*) >>1, there are more points at scale *r* than expected under a random distribution, which indicates an aggregated pattern at scale *r*. For the correction of edge effects, the translation correction method was used in the analysis [55].

We used the method of random-labeling null model within a case–control design [33] to study NDD on the two focal species from saplings to juveniles, where saplings and juveniles were used as cases (pattern *i*) and adults as controls (pattern *j*). In the case-control design, the control pattern is used to account for the large-scale habitat heterogeneity and intensity of seed rain [33], [34]. This method is based on two assumptions: 1) small-scale patterns are usually attributed to plant–plant interactions, whereas large-scale patterns are usually attributed to habitat heterogeneity [58] and 2) adult trees have undergone excessive thinning over time due to habitat heterogeneity and most likely represent those which lived in sites most favorable for the species. Therefore, the large-scale pattern (e.g. at *r* >10 m) of adults can be used as an indicator of environmental habitat preferences and can be used to control for the large-scale habitat heterogeneity [59], [60]. Under the null hypothesis of random-labeling, labels (case or control) are assigned to points randomly, conditioning the observed locations of the points in the joined patterns of cases and controls [61]. If the null hypothesis is rejected, the case-control approach may identify specific factors (i.e. NDD) other than habitat heterogeneity that may influence the spatial pattern of trees, by comparing the differences between case patterns and control patterns of trees in different life stages [18], [33]. The *g*(*r*) functions are invariant under random thinning of trees, hence we would expect *g_ii_*(*r*) = *g_jj_*(*r*) = *g_ij_*(*r*) = *g_ji_*(*r*). In this study, NDD is examined by using *g_ii_*(*r*) – *g_ij_*(*r*) as test statistics [33], [34], [62].

According to the prediction of NDD, tree survival would be low if individuals are growing in high density patches of conspecifics. Therefore, if there is strong NDD in saplings, the surviving juveniles would be less aggregated than saplings. To test this hypothesis, we studied the difference in the degree of aggregation between saplings and juveniles. We used *A_i_*(*r*) = *g_ii_*(*r*) – *g_ij_*(*r*) as the test statistic to determine whether cases *i* show an additional pattern that is independent of the controls *j*. If *A_i_*(*r*) >>0, cases can be said to exhibit additional aggregated patterns relative to adults, irrespective of whether habitat heterogeneity is present or not [62], [63]. The change in additional aggregation from saplings to juveniles at scale *r* can be expressed by the formula: *T*(*r*)  =  *A_saplings_*(*r*) – *A_juveniles_*(*r*). For a species, if *A_saplings_*(*r*) >>0 and *T*(*r*) >>0, we would infer that NDD existed in saplings. If this is the case, *T*(*r*) can be used to indicate the strength of NDD at different scales.

All spatial point pattern analyses were implemented with the “spatstat” package of R [64]. We focused on the scale of 0–20 m for the analyses above, assuming that tree-tree interactions could be efficiently indicated by this scale [65]. We performed 199 Monte Carlo simulations of the random labeling null model and used the 5th-lowest and 5th-highest values (i.e., extreme 0.25% simulated cases at either end) as simulation envelopes. However, this simulation inference yields an underestimated Type I error rate because the tests are performed at different concurrent scales [66]. We combined the simulation method with a goodness-of-fit test (GOF) [67]. Significant deviations were only determined for those data sets where the observed GOF's *P* value (*P*_GOF_) were less than 0.025 [58], [66].

## Results

### Diameter distributions

**Figure 1:**
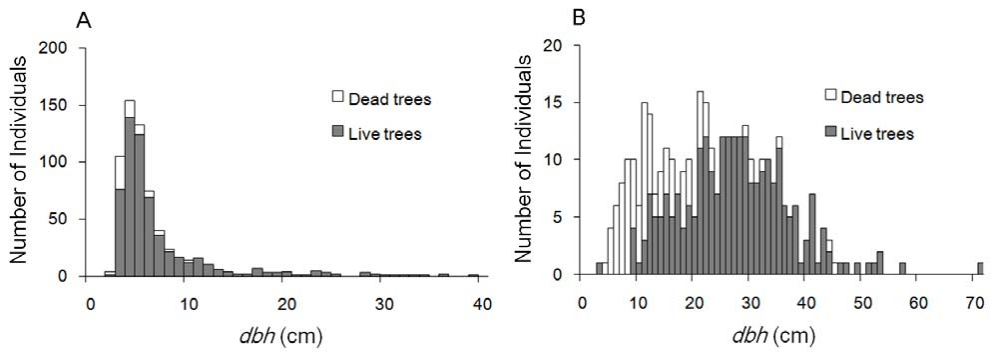
*Dbh* distributions of *Chamaecyparis obtusa* (a) and *Quercus serrata* (b). Live trees are those individuals that were alive in the 2011 census; dead trees are those individuals that were alive in the 2000 census but were found dead in the 2011 census.

The diameter distribution of CHOB was strongly right-skewed with 89% trees less than 10 cm *dbh* (Fig. 1a); this is a pattern typical of late-successional species that have a large number of suppressed young trees. In contrast, the diameter distribution of QUSE was symmetrical with a lack of small trees (Fig. 1b); this is a pattern typical of early-successional species that establish as approximately even-aged cohorts in large forest gaps. For both CHOB and QUSE, dead trees were concentrated in the smaller diameter classes (Fig. 1). Young trees of QUSE had a much greater mortality rate compared with CHOB (Fig. 1). For trees that were alive and <20 cm *dbh* in the year 2000 census, 50.1% of QUSE were dead in the year 2011 census; whereas, the mortality rate was only 10.8% for CHOB.

### Negative density dependence

**Figure 2:**
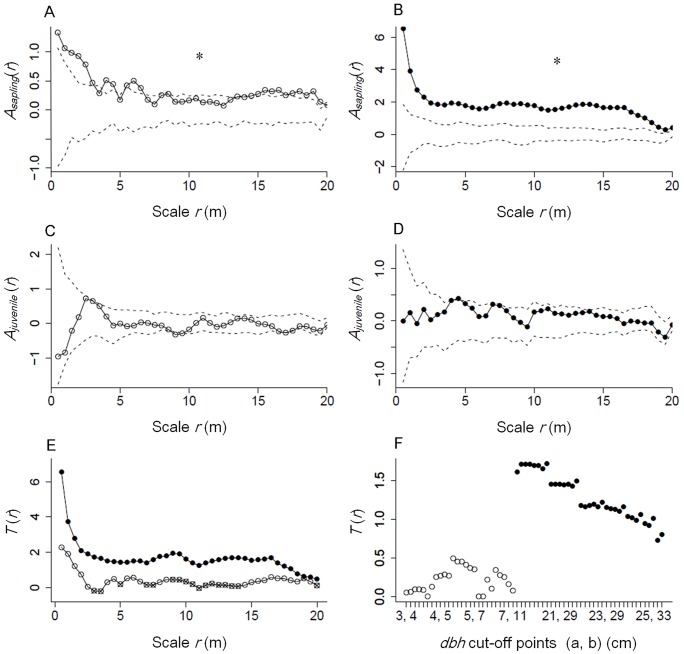
Changes of additional aggregation from saplings to juveniles. Open circles show the result of *Chamaecyparis obtusa* and filled circles show the result of *Quercus serrata*. The test statistic *A_j_*(*r*) (*A_saplings_*(*r*) or *A_juveniles_*(*r*)) evaluates if there is an additional aggregation within cases (saplings or juveniles) that is independent from adults. *A_j_*(*r*) >>0 means there is an additional aggregation within cases relative to adults. *A_j_*(*r*) ≈0 means patterns of cases and adults are created by the same stochastic process. The scale *r* is the radius around a focal tree of the pattern. Dotted lines were the 95% simulation envelopes constructed using the 5th-lowest and 5th-highest value of 199 Monte Carlo simulations of the null model. Comparison of the strength of NDD effect experienced by the two focal species (e) and Comparison of the strength of NDD experienced by the two focal species using all *dbh* cut-offs (f). *Dbh* cut-off points (a, b) are the cut-offs for defining life stages of trees: sapling (≥2 and <a), juvenile (≥a and <b) and adult (≥b). Units of cut-off points are centimeters. The cut-off point lists can be found in Table S1 and S2. *: significant difference by the goodness-of-fit test (*P_GOF_*<0.025).

For both CHOB and QUSE, saplings showed additional aggregated patterns relative to adults, as their test statistic *A_saplings_*(*r*) >>0 (*P_GOF_* = 0.005 for CHOB and QUSE) (Fig. 2a, b). For CHOB, significant deviations were found at the scale of 0–3, 4–4.5, 5.5–6.5, 8.5, 14–16.5, 17.5–19.5 m (Fig. 2a); for QUSE, significant deviations were found at all test scales (Fig. 2b). In juveniles, these aggregated patterns diminished for both species, as their test statistics demonstrate (*A_juveniles_*(*r*)≈0) (Fig. 2c, d). These results indicate that both focal species were influenced by NDD at the sapling stage. For both species, the decrease in additional aggregation was the greatest at the smallest scale and had a trend of decreasing with increasing scales (Fig. 2e), indicating that NDD occurred predominantly at close distances among neighbors. QUSE experienced stronger NDD in the sapling stage as *T*(*r*) is greater for QUSE compared with CHOB at all test scales (Fig. 2e). For all *dbh* cut-offs, the mean *T*(*r*) values were greater for QUSE than CHOB (Fig. 2f, Table S1 and S2), indicating the above finding was not influenced by the selection of different *dbh* cut-offs.

## Discussion

NDD has been shown to play an important role in the population dynamics of tree communities in numerous studies [1], [10], [31], [68]–[70]. Our study examined the spatial patterns and tested NDD for two abundant tree species with different shade tolerance at later life stages.

Plants are most susceptible to natural enemies and abiotic stresses in early life stages, therefore, most demographic winnowing of propagules takes place at early life stages [71]–[73]. Thus, one might expect NDD to be subdued at later life stages. However, recent studies pointed out that NDD also exists at later life stages [4], [15], [18], [22], [54], [74]. For example, Zhu et al. [18] studied 47 common tree species (*dbh* ≥ 1 cm) and found 39 of them exhibited conspecific density-dependent thinning in a subtropical forest by comparing the strength of aggregation from saplings to juveniles. Piao et al. [54] studied 15 common tree species (*dbh* ≥ 1 cm) in a temperate forest and found 11 of them showed conspecific density-dependent thinning, also by comparing the strength of aggregation from saplings to juveniles. Carson et al. [75] suggested that ecologists should focus on NDD occurring at later life stages in order to get a complete understanding of the effect in promoting species coexistence. In our later life stage study, we found NDD regulated both focal species. We also found NDD was the greatest at small scales and decreased with increasing scales for saplings. These results may be due to the decline in aggregation of saplings with increasing distance from parent trees, which had likely resulted from dispersal limitation [76]. Those findings are consistent with the distance-dependent model which predicts that survival is negatively correlated with the distance from the conspecific adult [77]. Distance dependence and NDD leads to a decreased probability that a species will replace itself locally and therefore promotes species coexistence [5], [77].

Shade-tolerant tree species have been shown to have better defenses against natural enemies than shade-intolerant tree species [42], [43]. Therefore, one could predict that shade-intolerant tree species experience greater NDD than shade-tolerant tree species. For example, Augspurger and Kelly [11] found that seedling disease susceptibility was negatively correlated with shade tolerance. Comita et al. [7] found that variations in species' shade tolerance could mask the negative relationship between seedling survival and abundance. Our result is consistent with the above prediction: we found that QUSE (a shade-intolerant and early-successional species) experienced greater NDD compared with CHOB (a shade-tolerant and late-successional species). Because this result can be caused by the great differences in the size class cut-offs between the two species, we used all possible size class cut-offs to implement the analyses and therefore our results are not likely caused by the specific size cut-offs selected. Our observation and previous studies suggest that attempting to quantify the contribution of NDD should take into account the variation in species' shade tolerance.

The stronger NDD experienced by QUSE may play an important role in the replacement of QUSE by other late-successional species such as CHOB, as self-thinning of early-successional trees could progressively open up the forest canopy and create open spaces that promote the survival of late-successional trees [20]. However, further studies should be implemented to examine whether the subsequent survival of late-successional trees such as CHOB are higher in those open spaces created by QUSE.

## Conclusions

Our study tested NDD for two abundant species found in the Keumsan LTER. We found NDD on the two abundant tree species at later life stages. We also found the shade-intolerant species suffered stronger NDD compared with the shade-tolerant species. Our findings highlighted the importance of testing NDD at later life stage for improved understanding of species coexistence. We also highlighted the importance of considering species shade tolerance in NDD study.

## Supporting Information

Table S1 **List of** ***dbh*** **cut-off points for defining life stages of** ***Chamaecyparis obtusa*** **: sapling (≥2 and <a), juvenile (≥a and <b) and adult (≥b).**
(DOCX)

Table S2 **List of** ***dbh*** **cut-off points for defining life stages of** ***Quercus serrata*** **: sapling (≥2 and <a), juvenile (≥a and <b) and adult (≥b).**
(DOCX)

## References

[1] Wright SJ (2002) Plant diversity in tropical forests: a review of mechanisms of species coexistence. Oecologia 130: 1–14. 28547014 10.1007/s004420100809

[2] Comita LS, Muller-Landau HC, Aguilar S, Hubbell SP (2010) Asymmetric density dependence shapes species abundances in a tropical tree community. Science (New York, NY) 329: 330–332. 10.1126/science.1190772 20576853

[3] Harms KE, Wright SJ, Calderón O, Hernández A, Herre EA (2000) Pervasive density-dependent recruitment enhances seedling diversity in a tropical forest. Nature 404: 493–495. 10761916 10.1038/35006630

[4] Peters HA (2003) Neighbour-regulated mortality: the influence of positive and negative density dependence on tree populations in species-rich tropical forests. Ecol Lett 6: 757–765.

[5] Queenborough SA, Burslem DFRP, Garwood NC, Valencia R (2007) Neighborhood and community interactions determine the spatial pattern of tropical tree seedling survival. Ecology 88: 2248–2258. 17918403 10.1890/06-0737.1

[6] Bai X, Queenborough SA, Wang X, Zhang J, Li B, et al (2012) Effects of local biotic neighbors and habitat heterogeneity on tree and shrub seedling survival in an old-growth temperate forest. Oecologia 170: 755–765. 22644047 10.1007/s00442-012-2348-2

[7] Comita LS, Hubbell SP (2009) Local neighborhood and species' shade tolerance influence survival in a diverse seedling bank. Ecology 90: 328–334. 19323215 10.1890/08-0451.1

[8] Queenborough SA, Burslem DFRP, Garwood NC, Valencia R (2009) Taxonomic scale-dependence of habitat niche partitioning and biotic neighbourhood on survival of tropical tree seedlings. Proceedings Biological sciences/The Royal Society 276: 4197–4205. 10.1098/rspb.2009.0921 PMC2821336 19740886

[9] Bell T, Freckleton RP, Lewis OT (2006) Plant pathogens drive density-dependent seedling mortality in a tropical tree. Ecol Lett 9: 569–574. 16643302 10.1111/j.1461-0248.2006.00905.x

[10] Liu X, Liang M, Etienne RS, Wang Y, Staehelin C, et al (2012) Experimental evidence for a phylogenetic Janzen-Connell effect in a subtropical forest. Ecol Lett 15: 111–118. 22082078 10.1111/j.1461-0248.2011.01715.x

[11] Augspurger CK, Kelly CK (1984) Pathogen mortality of tropical tree seedlings: experimental studies of the effects of dispersal distance, seedling density, and light conditions Oecologia. 61: 211–217. 10.1007/BF00396763 28309414

[12] Walsh RK, Bradley C, Apperson CS, Gould F (2012) An Experimental Field Study of Delayed Density Dependence in Natural Populations of *Aedes albopictus* . 7: e35959. 10.1371/journal.pone.0035959 PMC3338560 22563428

[13] Mangan SA, Schnitzer SA, Herre EA, Mack KML, Valencia MC, et al (2010) Negative plant-soil feedback predicts tree-species relative abundance in a tropical forest. Nature 466: 752–755. 20581819 10.1038/nature09273

[14] Condit R, Hubbell SP, Foster RB (1992) Recruitment Near Conspecific Adults and the Maintenance of Tree and Shrub Diversity in a Neotropical Forest. Am Nat 140: 261–286. 19426059 10.1086/285412

[15] Wills C, Condit R, Foster RB, Hubbell SP (1997) Strong density- and diversity-related effects help to maintain tree species diversity in a neotropical forest. Proc Natl Acad Sci U S A 94: 1252–1257. 11038601 10.1073/pnas.94.4.1252 PMC19777

[16] Uriarte M, Canham CD, Thompson J, Zimmerman JK (2004) A neighborhood analysis of tree growth and survival in a hurricane-driven tropical forest. Ecol Monogr 74: 591–614.

[17] Comita LS, Uriarte M, Thompson J, Jonckheere I, Canham CD, et al (2009) Abiotic and biotic drivers of seedling survival in a hurricane-impacted tropical forest. J Ecol 97: 1346–1359.

[18] Zhu Y, Mi X, Ren H, Ma K (2010) Density dependence is prevalent in a heterogeneous subtropical forest. Oikos 119: 109–119.

[19] Kenkel NC (1988) Pattern of self-thinning in jack pine: testing the random mortality hypothesis. Ecology 69: 1017–1024.

[20] He F, Duncan RP (2000) Density-dependent effects on tree survival in an old-growth Douglas fir forest. J Ecol 88: 676–688.

[21] Lambers JHR, Clark JS, Beckage B (2002) Density-dependent mortality and the latitudinal gradient in species diversity. Nature 417: 732–735. 12066182 10.1038/nature00809

[22] Zhang J, Hao Z, Sun I-F, Song B, Ye J, et al (2009) Density dependence on tree survival in an old-growth temperate forest in northeastern China. Ann For Sci 66: 204–204.

[23] Johnson DJ, Beaulieu WT, Bever JD, Clay K (2012) Conspecific negative density dependence and forest diversity. Science (New York, NY) 336: 904–907. 10.1126/science.1220269 22605774

[24] Ratikainen II, Gill JA, Gunnarsson TG, Sutherland WJ, Kokko H (2008) When density dependence is not instantaneous: theoretical developments and management implications. Ecol Lett 11: 184–198. 17979979 10.1111/j.1461-0248.2007.01122.x

[25] Webster MS, Marra PP, Haig SM, Bensch S, Holmes RT (2002) Links between worlds: unraveling migratory connectivity. Trends Ecol Evol 17: 76–83.

[26] Nathan R, Safriel UN, Noy-Meir I, Schiller G (2000) Spatiotemporal variation in seed dispersal and recruitment near and far from *Pinus halepensis* trees. Ecology 81: 2156–2169.

[27] Howe HF, Miriti MN (2004) When Seed Dispersal Matters. BioOne 54: 651–660.

[28] Schupp EW, Jordano P (2011) The full path of Janzen-Connell effects: genetic tracking of seeds to adult plant recruitment. Mol Ecol 20: 3953–3955. 21951419 10.1111/j.1365-294X.2011.05202.x

[29] Lan GY, Zhu H, Cao M, Hu YH, Wang H, et al (2009) Spatial dispersion patterns of trees in a tropical rainforest in Xishuangbanna, southwest China. Ecol Res 24: 1117–1124.

[30] Comita LS, Condit R, Hubbell SP (2007) Developmental changes in habitat associations of tropical trees. J Ecol 95: 482–492.

[31] Paine CET, Norden N, Chave J, Forget P-M, Fortunel C, et al (2012) Phylogenetic density dependence and environmental filtering predict seedling mortality in a tropical forest. Ecol Lett 15: 34–41. 22004454 10.1111/j.1461-0248.2011.01705.x

[32] Grubb PJ (1977) The maintenance of species-richness in plant communities: the importance of the regeneration niche. BRCPS 52: 107–145.

[33] Getzin S, Wiegand T, Wiegand K, He F (2008) Heterogeneity influences spatial patterns and demographics in forest stands. J Ecol 96: 807–820.

[34] Luo Z, Mi X, Chen X, Ye Z, Ding B (2012) Density dependence is not very prevalent in a heterogeneous subtropical forest. Oikos 121: 1239–1250.

[35] Hubbell SP (2001) Local neighborhood effects on long-term survival of individual trees in a neotropical forest. Ecol Res 72: 35–875.

[36] Hubbell SP (1979) Tree Dispersion, Abundance, and Diversity in a Tropical Dry Forest. Science 203: 1299–1309. 17780463 10.1126/science.203.4387.1299

[37] Moeur M (1997) Spatial models of competition and gap dynamics in old-growth plicata forests. For Ecol Manage 94: 175–186.

[38] Beckage B, Clark JS (2003) Seedling survival and growth of three forest tree species: The role of spatial heterogeneity. Ecology 84: 1849–1861.

[39] Harper JL (1977) Population biology of plants. London, UK: Academic Press.

[40] Augspurger CK, Kelly CK (1984) Pathogen mortality of tropical tree seedlings: experimental studies of the effects of dispersal distance, seedling density, and light conditions. Oecologia 61: 211–217. 28309414 10.1007/BF00396763

[41] Bachelot B, Kobe RK (2013) Rare species advantage? Richness of damage types due to natural enemies increases with species abundance in a wet tropical forest. J Ecol 101: 846–856.

[42] Kobe RK, Vriesendorp CF (2011) Conspecific density dependence in seedlings varies with species shade tolerance in a wet tropical forest. Ecol Lett 14: 503–510. 21429063 10.1111/j.1461-0248.2011.01612.x

[43] Coley PD, Barone JA (1996) Herbivory and plant defenses in tropical forests. Annual Review of Ecology, Evolution, and Systematics 27: 305–335.

[44] Kitajima K, Poorter L (2010) Tissue-level leaf toughness, but not lamina thickness, predicts sapling leaf lifespan and shade tolerance of tropical tree species. New Phytol 168: 708–721. 10.1111/j.1469-8137.2010.03212.x 20298481

[45] Kobe RK (1997) Carbohydrate allocation to storage as a basis of interspecific variation in sapling survivorship and growth. Oikos 80: 226–233.

[46] Myers JA, Kitajima K (2007) Carhohydrate storage enhances seedling shade and stress tolerance in a neotropical forest. J Ecol 95: 383–395.

[47] McCarthy-Neumann S, Kobe RK (2008) Tolerance of soil pathogens co-varies with shade tolerance across species of tropical tree seedlings. Ecology 89: 1883–1892. 18705375 10.1890/07-0211.1

[48] Korea Meteorological Administration (2012) Available: http://www.kma.go.kr.

[49] Bae JS, Joo RW, Kim YS (2012) Forest transition in South Korea: reality, path and drivers. Land Use Policy 29: 198–207.

[50] Kim C, Lim JH, Lee IK, Park BB, Chun JH (2013) Annual variations of litterfall production in a broadleaved deciduous forest at the Mt. Keumsan LTER site. Journal of Korean Forest Society 102: 210–215.

[51] Fujii S, Kasuya N (2008) Fine root biomass and morphology of Pinus densiflora under competitive stress by *Chamaecyparis obtusa* . J For Res 13: 185–189.

[52] Fujimaki R, McGonigle TP, Takeda H (2004) Soil micro-habitat effects on fine roots of Chamaecyparis obtusa Endl.: a field experiment using root ingrowth cores. Plant Soil 266: 325–332.

[53] Seiwa K, Kikuzawa K, Kadowaki T, Akasaka S, Ueno N (2006) Shoot lifespan in relation to successional status in deciduous broad-leaved tree species in a temperate forest. New Phytol 169: 537–548. 16411956 10.1111/j.1469-8137.2005.01608.x

[54] Piao T, Comita LS, Jin G, Kim JH (2013) Density dependence across multiple life stages in a temperate old-growth forest of northeast China. Oecologia 172: 207–217. 23053238 10.1007/s00442-012-2481-y PMC3627022

[55] Stoyan D, Stoyan H (1994) Fractals, random shapes, and point fields: methods of geometrical statistics. Wiley, Chichester.

[56] Illian J, Penttinen A, Stoyan H, Stoyan D (2008) Statistical analysis and modelling of spatial point patterns: Wiley, London.

[57] Ripley BD (1976) The second-order analysis of stationary point processes. J Appl Prob 13: 255–266. J Appl Probab 13: 255–266.

[58] Wiegand T, Gunatilleke S, Gunatilleke N (2007) Species associations in a heterogeneous Srilankan dipterocarp forest. Am Nat 170: E77–95. 17891727 10.1086/521240

[59] Stoyan D, Penttinen A (2000) Recent applications of point process methods in forestry statistics. Statistical Science 15: 61–78.

[60] Condit R, Ashton PS, Baker P, Bunyavejchewin S, Gunatilleke S, et al (2000) Spatial patterns in the distribution of tropical tree species. Science (New York, NY) 288: 1414–1418. 10.1126/science.288.5470.1414 10827950

[61] Wiegand T, Moloney KA (2004) Rings, circles and nullmodels for point pattern analysis in ecology. Oikos 104: 209–229.

[62] Getzin S, Dean C, He F, Trofymow JA, Wiegand K, et al (2006) Spatial patterns and competition of tree species in a Douglas-fir chronosequence on Vancouver Island. Ecography 29: 671–682.

[63] Watson DM, Roshier DA, Wiegand T (2007) Spatial ecology of a parasitic shrub: patterns and predictions. Austral Ecol 32: 359–369.

[64] R Development Core Team (2011) R: a language and environment for statistical computing. R foundation for statistical computing, Vienna. ISBN 3-900051-07-0. Available: http://www.R-project.org. Accessed 2011 April 13.

[65] McCarthy-Neumann S, Kobe RK (2010) Conspecific plant–soil feedbacks reduce survivorship and growth of tropical tree seedlings. J Ecol 98: 396–407.

[66] Loosmore NB, Ford ED (2006) Statistical inference using the G or K point pattern spatial statistics. Ecology 87: 1925–1931. 16937629 10.1890/0012-9658(2006)87[1925:siutgo]2.0.co;2

[67] Diggle P (2003) Statistical analysis of spatial point patterns. Arnold, London.

[68] Chesson P (2000) Mechanisms of maintenance of species diversity. Annu Rev Ecol Syst 31: 343–366.

[69] Volkov I, Banavar JR, He F, Hubbell SP, Maritan A (2005) Density dependence explains tree species abundance and diversity in tropical forests. Nature 438: 658–661. 16319890 10.1038/nature04030

[70] Yamazaki M, Iwamoto S, Seiwa K (2009) Distance- and density-dependent seedling mortality caused by several diseases in eight tree species co-occurring in a temperate forest. Plant Ecol 201: 181–196.

[71] Terborgh J (2012) Enemies maintain hyperdiverse tropical forests. Am Nat 179: 303–314. 22322219 10.1086/664183

[72] Connell JH (1971) On the role of natural enemies in preventing competitive exclusion in some marine animals and in rain forest trees. In: den Boer PJ, Gradwell GR, editors. Dynamics of populations: Centre for Agricultural Publications and Documentation, Wageningen. pp. 298–310.

[73] Hyatt LA, Rosenberg MS, Howard TG, Bole G, Fang W, et al (2003) The distance dependence prediction of the Janzen-Connell hypothesis: a meta-analysis. Oikos 103: 590–602.

[74] Stoll P, Newbery DM (2005) Evidence of species-specific neighborhood effects in the Dipterocarpaceae of a Bornean rain forest. Ecology 86: 3048–3062. 10.1890/13-0366.1 24597229

[75] Carson WP, Anderson J, Leigh E, Schnitzer SA (2008) Challenges Associated with Testing and Falsifying the Janzen-Connell Hypothesis: A Review and Critique. In: Carson WP, Schnitzer SA, editors. Tropical Forest Community Ecology: Blackwell Publishing, Oxford. pp. 210–241.

[76] He F, Legendre P, LaFrankie JV (1997) Distribution patterns of tree species in a Malaysian tropical rain forest. Journal of Vegetation Science 8: 105–114.

[77] Janzen DH (1970) Herbivores and the Number of Tree Species in Tropical Forests. Am Nat 104: 501–528.

