# Supplementary material for: Negative Density Dependence Regulates Two Tree Species at Later Life Stage in a Temperate Forest
Source: PLoS One. 2014 Jul 24;9(7):e103344. doi: 10.1371/journal.pone.0103344 (PMC4110017; doi:10.1371/journal.pone.0103344)
Supplement: Table S1 — List of dbh cut-off points for defining life stages of Chamaecyparis obtusa : sapling (≥2 and <a), juvenile (≥a and <b) and adult (≥b). (DOCX) [file pone.0103344.s001.docx]

**Table S1.** List of *dbh* cut-off points for defining life stages of *Chamaecyparis obtusa*: sapling (≥ 2 and < a), juvenile (≥ a and < b) and adult (≥ b). Mean *T*(*r*) is the mean value of change of additional aggregation for testing the density-dependent effect from saplings to juveniles at scales 0-20 m.

| Cut-off points (a, b) (cm) | Sapling | Juvenile | Adult | Mean *T*(*r*) |
| --- | --- | --- | --- | --- |
| 3, 4 | 177 | 196 | 383 | 0.05 |
| 3, 5 | 177 | 330 | 249 | 0.06 |
| 3, 6 | 177 | 404 | 175 | 0.10 |
| 3, 7 | 177 | 441 | 138 | 0.09 |
| 3, 8 | 177 | 464 | 115 | 0.09 |
| 3, 9 | 177 | 485 | 94 | - |
| 3, 10 | 177 | 497 | 82 | 0.13 |
| 3, 11 | 177 | 509 | 70 | 0.25 |
| 4, 5 | 373 | 134 | 249 | 0.27 |
| 4, 6 | 373 | 208 | 175 | 0.28 |
| 4, 7 | 373 | 245 | 138 | 0.27 |
| 4, 8 | 373 | 268 | 115 | 0.50 |
| 4, 9 | 373 | 289 | 94 | 0.45 |
| 4, 10 | 373 | 301 | 82 | 0.45 |
| 4, 11 | 373 | 313 | 70 | 0.40 |
| 5, 6 | 507 | 74 | 175 | 0.37 |
| 5, 7 | 507 | 111 | 138 | 0.35 |
| 5, 8 | 507 | 134 | 115 | - |
| 5, 9 | 507 | 155 | 94 | - |
| 5, 10 | 507 | 167 | 82 | 0.21 |
| 5, 11 | 507 | 179 | 70 | 0.10 |
| 6, 8 | 581 | 60 | 115 | 0.34 |
| 6, 9 | 581 | 81 | 94 | 0.27 |
| 6, 10 | 581 | 93 | 82 | 0.25 |
| 6, 11 | 581 | 105 | 70 | 0.16 |
| 7, 11 | 618 | 68 | 70 | 0.08 |
