# Supplementary material for: Negative Density Dependence Regulates Two Tree Species at Later Life Stage in a Temperate Forest
Source: PLoS One. 2014 Jul 24;9(7):e103344. doi: 10.1371/journal.pone.0103344 (PMC4110017; doi:10.1371/journal.pone.0103344)
Supplement: Table S2 — List of dbh cut-off points for defining life stages of Quercus serrata : sapling (≥2 and <a), juvenile (≥a and <b) and adult (≥b). (DOCX) [file pone.0103344.s002.docx]

**Table S2.** List of *dbh* cut-off points for defining life stages of *Quercus serrata*: sapling (≥ 2 and < a), juvenile (≥ a and < b) and adult (≥ b). Mean *T*(*r*) is the mean value of change of additional aggregation for testing the density-dependent effect from saplings to juveniles at scales 0-20 m.

| Cut-off points (a, b) (cm) | Sapling | Juvenile | Adult | Mean *T*(*r*) |
| --- | --- | --- | --- | --- |
| 20, 26 | 60 | 62 | 135 | 1.61 |
| 20, 27 | 60 | 74 | 123 | 1.71 |
| 20, 28 | 60 | 87 | 110 | 1.71 |
| 20, 29 | 60 | 96 | 101 | 1.71 |
| 20, 30 | 60 | 106 | 91 | 1.69 |
| 20, 31 | 60 | 114 | 83 | 1.70 |
| 20, 32 | 60 | 124 | 73 | 1.65 |
| 20, 33 | 60 | 133 | 64 | 1.72 |
| 21, 27 | 70 | 64 | 123 | 1.45 |
| 21, 28 | 70 | 77 | 110 | 1.46 |
| 21, 29 | 70 | 86 | 101 | 1.45 |
| 21, 30 | 70 | 96 | 91 | 1.44 |
| 21, 31 | 70 | 104 | 83 | 1.45 |
| 21, 32 | 70 | 114 | 73 | 1.42 |
| 21, 33 | 70 | 123 | 64 | 1.49 |
| 22, 28 | 82 | 65 | 110 | 1.18 |
| 22, 29 | 82 | 74 | 101 | 1.16 |
| 22, 30 | 82 | 84 | 91 | 1.17 |
| 22, 31 | 82 | 92 | 83 | 1.19 |
| 22, 32 | 82 | 102 | 73 | 1.16 |
| 22, 33 | 82 | 111 | 64 | 1.22 |
| 23, 29 | 91 | 65 | 101 | 1.15 |
| 23, 30 | 91 | 75 | 91 | 1.13 |
| 23, 31 | 91 | 83 | 83 | 1.13 |
| 23, 32 | 91 | 93 | 73 | 1.10 |
| 23, 33 | 91 | 102 | 64 | 1.16 |
| 24, 30 | 98 | 68 | 91 | 1.03 |
| 24, 31 | 98 | 76 | 83 | 1.02 |
| 24, 32 | 98 | 86 | 73 | 0.98 |
| 24, 33 | 98 | 95 | 64 | 1.06 |
| 25, 31 | 111 | 63 | 83 | 0.94 |
| 25, 32 | 111 | 73 | 73 | 0.92 |
| 25, 33 | 111 | 82 | 64 | 1.01 |
| 26, 32 | 122 | 62 | 73 | 0.73 |
| 26, 33 | 122 | 71 | 64 | 0.80 |
